# Advances in external therapies of traditional Chinese medicine for the management of hyperuricemia: a comprehensive review

**DOI:** 10.3389/fendo.2025.1667523

**Published:** 2025-12-19

**Authors:** Minyang He, Nannan Jiang, Lin Jiang, Tingting Tang, Qiyun Zhang, Qichang Xu, Songze Li, Feng Zhang, Xiangcheng Fan, Jichun Han

**Affiliations:** 1 College of Traditional Chinese Medicine, Binzhou Medical University, Yantai, China; 2 Department of Gastroenterology, The Fourth Affiliated Hospital of School of Medicine, and International School of Medicine, International Institutes of Medicine, Zhejiang University, Yiwu, China; 3 Department of Anesthesiology, The Affiliated Yantai Yuhuangding Hospital of Qingdao University, Yantai, China; 4 Department of Pharmacy, Changzheng Hospital, Naval Medical University, Shanghai, China; 5 Tongde Hospital of Zhejiang Province Affiliated to Zhejiang Chinese Medical University (Tongde Hospital of Zhejiang Province), Hangzhou, Zhejiang, China; 6 Zhejiang Academy of Traditional Chinese Medicine, Hangzhou, Zhejiang, China

**Keywords:** hyperuricemia, gout, external therapy of traditional Chinese medicine, acupuncture, bloodletting

## Abstract

Hyperuricemia is a prevalent metabolic disorder whose rising incidence over recent years has been closely linked to the development of gout, renal dysfunction, and cardiovascular disease, thereby exerting a substantial burden on patient quality of life. External therapies of Traditional Chinese Medicine (TCM)-including acupuncture, *tuina* (therapeutic massage), bloodletting, and topical herbal applications-have been practiced for centuries and encompass a diverse array of modalities. These interventions exert their therapeutic effects by modulating meridian flow, regulating *qi* and *blood* circulation, and harmonizing visceral function, which collectively promote uric acid excretion, suppress its production, and alleviate clinical manifestations of hyperuricemia. However, the heterogeneity of techniques and the complexity of underlying mechanisms pose challenges to systematic evaluation. In this review, we critically summarize current evidence on the mechanistic basis of various TCM external therapies for hyperuricemia and document their efficacy in symptom improvement. By integrating pharmacological insights and clinical outcomes, we aim to provide a comprehensive theoretical framework to guide future research and optimize the application of external TCM therapies in managing hyperuricemia.

## 1 Introduction

Hyperuricemia is a metabolic disease that leads to the increase of serum uric acid (SUA) level due to excessive uric acid production or insufficient excretion in the body (1–3). Under normal circumstances, uric acid is the final product of purine metabolism, which is mainly excreted by the kidney. When the serum uric acid concentration exceeds 7.0 mg/dL, hyperuricemia can be diagnosed (4). The etiology of hyperuricemia is complex, which can be divided into primary and secondary types (5). Primary hyperuricemia is usually related to genetic factors, and the synthesis and degradation of purine in the body are abnormal, leading to excessive production of uric acid (6). Secondary hyperuricemia can be caused by many factors, such as renal insufficiency (7). Metabolic diseases such as diabetes (8), High purine diet (9), and adverse drug reactions (10). Importantly, hyperuricemia is not only a simple metabolic abnormality, but also related to gout (11), kidney calculi (12), Cardiovascular diseases (13) and metabolic syndrome (14) and other diseases are closely related. The accumulation of high uric acid can lead to the deposition of urate crystals in joints, kidneys and other tissues, which will lead to inflammatory reaction and further aggravate the disease (15).

At present, commonly used drugs for reducing uric acid in clinic are mainly divided into two categories. One category includes drugs that inhibit uric acid production represented by allopurinol, which can reduce uric acid production by inhibiting xanthine oxidase (16). Another category includes drugs that promote uric acid excretion, such as benbromarone, which can increase uric acid excretion by inhibiting renal tubular reabsorption of uric acid (17). In the acute attack of gout, in order to relieve joint pain, swelling and other symptoms, non-steroidal anti-inflammatory drugs, colchicine or glucocorticoid are often used to control inflammation (18). In addition, there are daily lifestyle interventions, including low-purine diet, avoiding eating high-purine foods such as animal offal, seafood and alcohol; Drink plenty of water, and the daily drinking amount is not less than 2000ml, so as to promote uric acid excretion (19, 20). For the treatment of hyperuricemia, although modern medicine has made some progress in drug treatment, it still faces problems such as side effects and drug dependence (21). In parallel with efficacy, the safety profiles of first-line urate-lowering and anti-inflammatory agents warrant emphasis. Among xanthine-oxidase inhibitors, allopurinol requires renal dose adjustment and carries a risk of allopurinol hypersensitivity syndrome (AHS) (22), whereas febuxostat has a reported cardiovascular risk signal, calling for caution in patients with established cardiovascular disease (23). Regarding uricosurics, probenecid increases urinary urate and the risk of nephrolithiasis and is unsuitable in significant renal impairment (24); lesinurad can cause renal adverse events and should be prescribed only in combination with a xanthine-oxidase inhibitor (25). For acute flares, individualized risk–benefit assessment is required because each agent carries distinct adverse effects: nonsteroidal anti-inflammatory drugs(NSAIDs) may cause gastrointestinal bleeding as well as cardiovascular and renal toxicity; colchicine can lead to GI intolerance and dose-related toxicity, including myopathy when combined with CYP3A4 or P-gp inhibitors; short courses of glucocorticoids increase the risk of hyperglycemia, hypertension, and infection (26). These safety considerations contextualize pharmacotherapy risks and motivate our parallel discussion of external TCM therapies as potential adjuncts or alternatives in selected patients. At the same time, because hyperuricemia needs long-term treatment and diet control, the quality of life of patients with hyperuricemia has seriously declined. Additionally, these patients often endure significant psychological distress (27). The external therapy of TCM has become an auxiliary treatment method worthy of attention because of its mild and safe characteristics.

**Figure 1:**
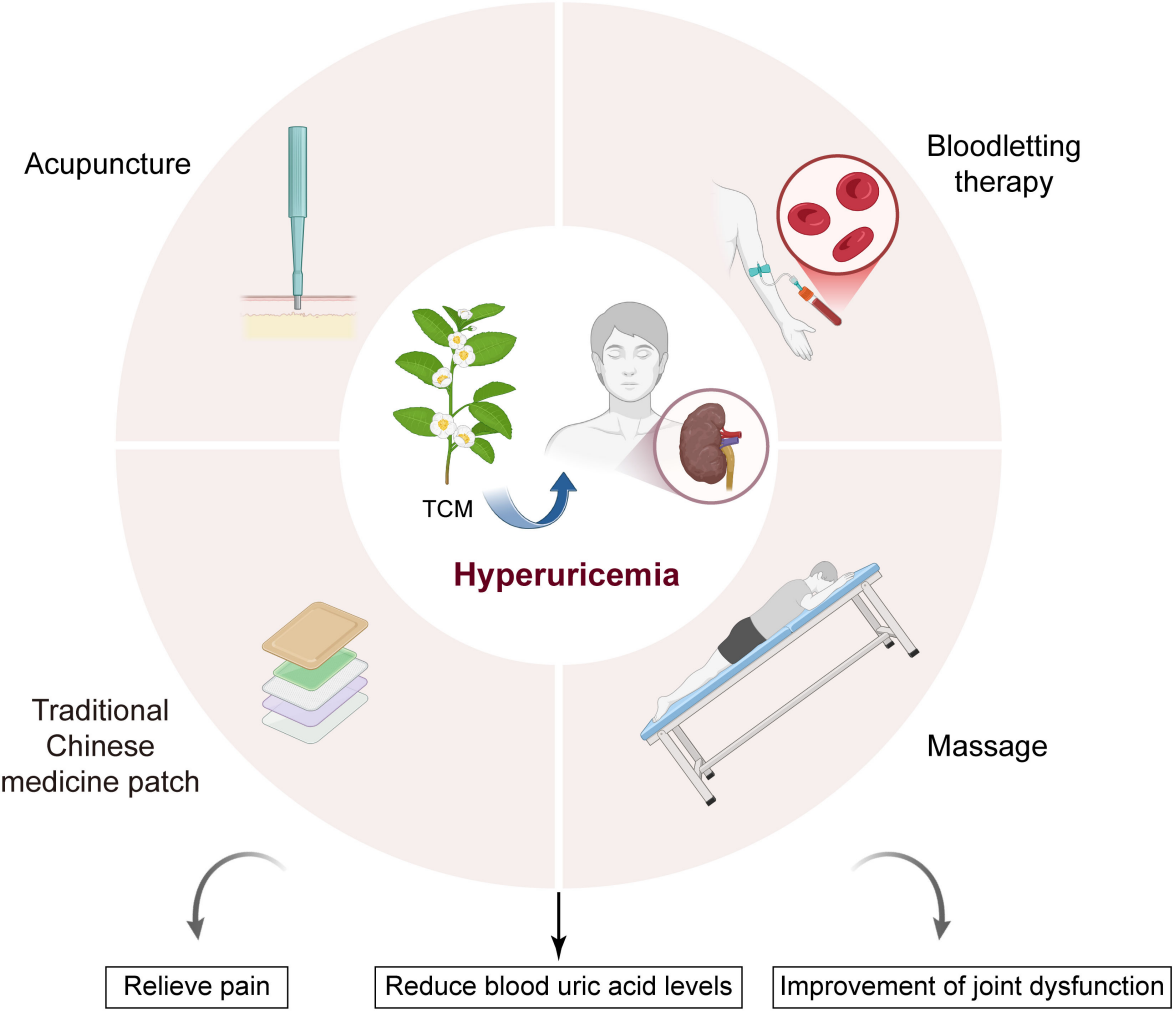
Treatment of hyperuricemia with external therapies of TCM. External TCM treatments, including acupuncture, massage, bloodletting therapy, and TCM patches, play a role in treating hyperuricemia by relieving pain, reducing blood uric acid levels, and improving joint dysfunction.

As an important part of TCM, external therapy of TCM has gradually formed a unique theoretical system and practical methods after thousands of years of development (28). TCM emphasizes “syndrome differentiation and treatment”, that is, according to the patient’s specific condition, physique and environmental factors, a personalized treatment plan is formulated (29). Different from the local treatment and symptomatic medication in modern medicine, the external therapy of TCM pays more attention to the overall conditioning of patients, and achieves the purpose of treating diseases by regulating *qi* and *blood*, balancing yin and yang, and enhancing the self-repair ability of the body (30). external therapy of TCM has a wide range of clinic external therapy applications, including but not limited to pain management, conditioning of functional diseases, endocrine disorders, metabolic syndrome and so on (31). External therapies such as acupuncture, bloodletting, massage and external therapy of TCM have been widely used in these fields, and many studies have proved that they have remarkable effects in relieving symptoms and improving patients’ quality of life (32). Especially in the treatment of hyperuricemia, these external therapies can help patients effectively reduce uric acid levels and relieve pain by promoting metabolism, improving blood circulation and regulating endocrine (**Figure 1**).

This paper summarizes the latest research progress of external therapy of TCM, such as acupuncture, bloodletting, massage and external therapy of TCM, and summarizes the mechanism and therapeutic effect of these external therapies in treating hyperuricemia in clinic, hoping to provide more specific theoretical basis for the application of external therapy of TCM in hyperuricemia, so as to improve new treatment schemes for patients with hyperuricemia.

## 2 Methods and strategies

### 2.1 Literature search strategy

A comprehensive literature search was conducted using four electronic databases: PubMed, Web of Science, China National Knowledge Infrastructure (CNKI), and Wanfang Data. Publications available up to January 2025 were considered. The search was limited to studies published in English or Chinese.

The following search terms and their synonyms were used: “hyperuricemia”, “gout”, “external therapy of TCM”, “acupuncture”, “bloodletting”, “tuina (massage)”, “fire needle”, and “topical herbal application”. Boolean operators (“AND”, “OR”) were used to optimize retrieval across various combinations.

### 2.2 Inclusion criteria

Studies were selected according to the following inclusion criteria: (1) Population: Adults diagnosed with hyperuricemia or gout; (2) Interventions: External TCM therapies including but not limited to acupuncture, electroacupuncture, bloodletting, pricking-cupping, tuina massage, fire needle, and topical herbal formulations; (3) Comparators: Conventional pharmacotherapy, placebo/sham interventions, usual care, or baseline controls; (4) Outcomes: At least one of the following—serum uric acid levels, pain intensity (e.g., VAS/NRS), joint swelling/function, gout flare frequency, inflammatory markers (e.g., CRP, ESR), renal function indices, or adverse events; (5) Study types: Randomized controlled trials (RCTs), quasi-randomized trials, cohort studies, case–control studies, and relevant systematic reviews or meta-analyses.

### 2.3 Exclusion criteria

The following studies were excluded: Editorials, letters, conference abstracts without full data, and non–peer-reviewed materials; Case reports or case series with fewer than 10 participants; Studies unrelated to external TCM therapy (e.g., oral decoctions only) or not focused on hyperuricemia/gout; Duplicated publications or studies with irreconcilable or missing outcome data; Pediatric-only populations when adult data were unavailable.

### 2.4 Study selection and data extraction

Two reviewers independently screened the titles, abstracts, and full texts of the retrieved articles. Disagreements were resolved by consensus through discussion. Relevant data were extracted, including: Study characteristics (author, year, country); Participant demographics and diagnostic criteria; Details of interventions and comparators; Reported outcomes and adverse events. The evidence was synthesized narratively, and no quantitative meta-analysis was conducted due to expected clinical and methodological heterogeneity among studies.

## 3 Treatment of hyperuricemia by external therapy of TCM

**Figure 2:**
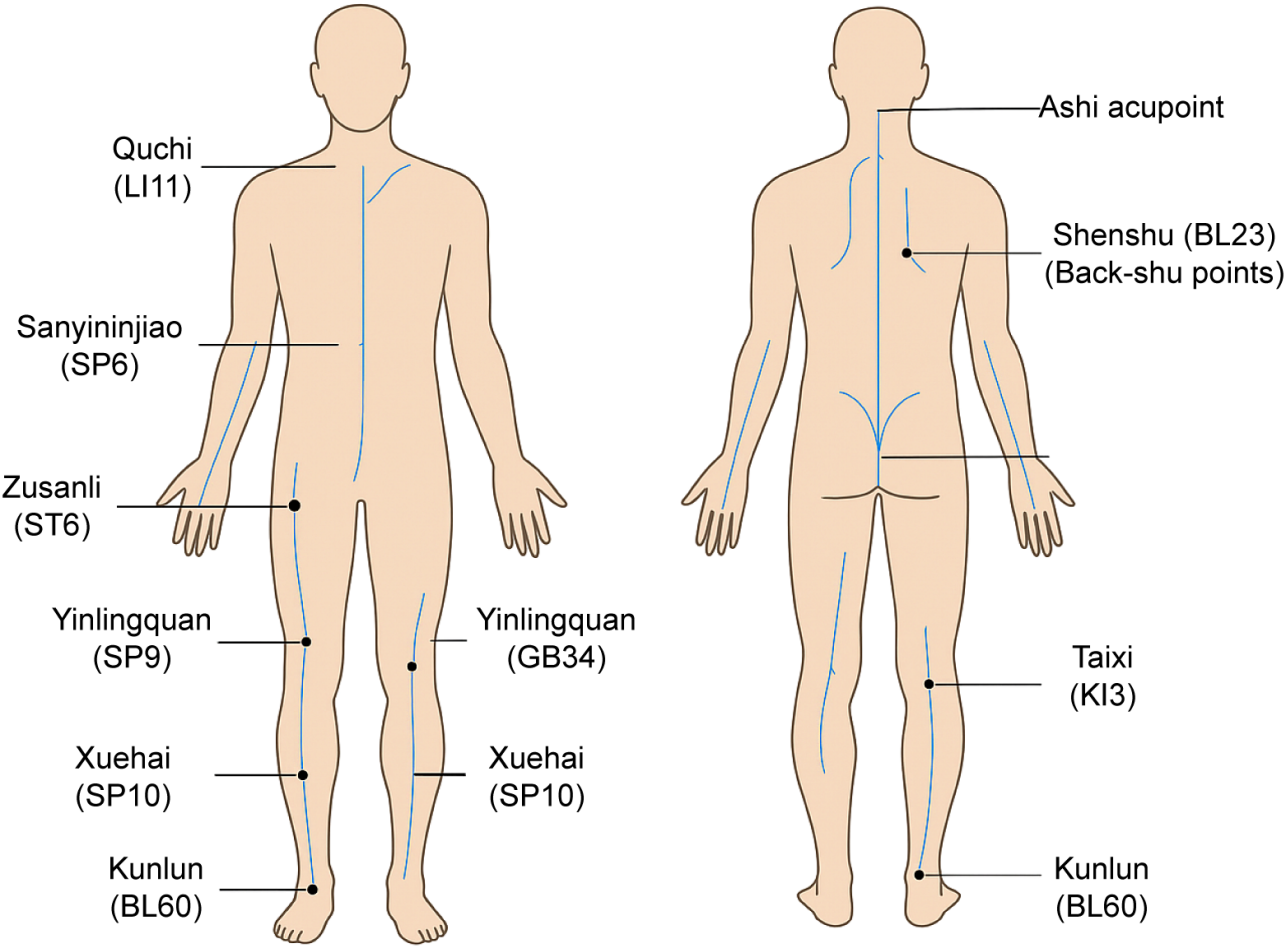
Illustration of commonly used acupoints in TCM external therapies for hyperuricemia and gout. The figure displays front and back views of a human body with selected acupuncture and pressure points labeled using their standard names and World Health Organization (WHO) meridian codes. Key points include Zusanli (ST36), Sanyinjiao (SP6), Yinlingquan (SP9), Xuehai (SP10), Yanglingquan (GB34), Quchi (LI11), Taixi (KI3), Weizhong (BL40), Shenshu (BL23), Kunlun (BL60), and Ashi points.

The main acupoints involved in external therapies for hyperuricemia are illustrated in **Figure 2** to enhance anatomical clarity and facilitate clinical reference.

The increasing prevalence of hyperuricemia and its complications has brought renewed interest to external therapies of TCM. While Western pharmacotherapy plays a central role in urate control, its limitations—including adverse effects, poor tolerance, and long-term dependency—highlight the need for complementary strategies (33, 34). In this section, we discuss TCM external therapies such as acupuncture, massage (*tuina*), bloodletting, and topical herbal applications (35). These treatments are guided by TCM principles such as *syndrome differentiation* and the regulation of *qi* (*vital energy*) and blood, while their physiological effects are increasingly interpreted through biomedical frameworks.

### 3.1 The basic theory of external therapy of TCM

In TCM theory, *qi* (*vital energy*) and blood are considered fundamental to sustaining physiological function and health. *Qi* serves as the driving force behind various bodily processes, while blood provides nourishment to tissues. When *qi* and blood flow freely through the meridians (energy channels), the body maintains homeostasis. However, modern lifestyle factors such as poor diet, stress, and sedentary habits can lead to *qi* stagnation and *blood stasis*, contributing to metabolic disorders such as hyperuricemia.

In TCM theory, *qi* (*vital energy*) and blood are considered fundamental to sustaining physiological function and health. *Qi* serves as the driving force behind various bodily processes, while blood provides nourishment to tissues (36). *Qi*, usually understood as the “*qi* of life”, is the driving force to promote various physiological activities in the body, while blood is the carrier of nutrients and undertakes the task of nourishing the whole body (37). The generation and application of *qi* and *blood* is one of the cores of TCM theory. In a healthy state, *qi* and *blood* are full, *qi* flows smoothly, and various systems of the body can operate in harmony, forming a good physiological state (38). However, in modern society, due to the change of lifestyle, unreasonable diet structure and the increase of psychological pressure, many people are facing the problem of imbalance between *qi* and *blood* (39). Hyperuricemia is a health problem closely related to the imbalance of *qi* and *blood*. In the theory of TCM, In TCM theory, hyperuricemia is considered a manifestation of damp-heat or *qi* stagnation and *blood* stasis—patterns that describe internal imbalance and impaired metabolic waste clearance. These correspond to inflammation, poor circulation, and metabolic dysfunction in biomedical terms (40). The accumulation of metabolic waste in the body is caused by damp heat, which affects the excretion of uric acid; *qi* stagnation may lead to poor blood circulation, and then affect the metabolism of uric acid. Therefore, it is particularly important to regulate *qi* and *blood* and dredge *meridians* for the treatment of hyperuricemia (41). In TCM, “dredging the *meridians*” refers to restoring the unobstructed flow of *qi* and *blood* through specific pathways in the body. It is believed to eliminate pathogenic factors, relieve pain, and normalize internal organ function by stimulating acupoints to open blocked channels. Biomedically, this may correspond to enhanced circulation, anti-inflammatory modulation, and neuromuscular regulation (42).

External therapies of TCM, such as acupuncture, massage and bloodletting, all achieve therapeutic purposes by regulating *qi* and *blood* and dredging *meridians* (43). Acupuncture stimulates specific acupoints to promote the flow of *qi* (vital energy) and blood, which in TCM theory supports the body’s self-regulation and repair. Biomedically, this may correspond to enhanced neurovascular activation and modulation of immune-inflammatory responses (44); Massage acts on muscles and *meridians* through manipulation to improve local blood circulation and relieve pain (45); Bleeding can expel damp-heat and blood stasis accumulated in the body through acupuncture bleeding, and promote human metabolism (46). On the whole, the theory of *qi* and *blood* plays an important guiding role in the external therapy of TCM. By regulating the state of *qi* and *blood*, the clinical symptoms of patients with hyperuricemia can be effectively improved, the excretion of uric acid can be promoted, and the patients can be helped to recover (47).

### 3.2 Acupuncture

Acupuncture, as an important part of external therapy of TCM, its basic principle and mechanism of action are of great significance in the treatment of hyperuricemia (48). It can regulate the flow of *qi* and *blood*, promote metabolism and achieve the effect of improving diseases by stimulating specific acupoints (49, 50). Meta-analysis shows that acupuncture has a good effect in treating hyperuricemia, and its analgesic effect is very significant (51, 52). TCM believes that hyperuricemia is related to spleen, liver and kidney. Acupuncture treatment often combines whole acupoint selection with local acupoint selection, and the main points commonly used are Foot Taiyin Spleen Meridian, Foot Yangming Stomach Meridian and Foot Jueyin Liver Meridian (53).

For asymptomatic hyperuricemia, acupuncture is primarily configured to lower serumuric acid and optimize renal urate handling. Acupuncture has a good therapeutic effect on asymptomatic hyperuricemia patients (54). Clinical studies have confirmed that acupuncture at five points on both sides of spleen meridian can increase urine volume and urine pH value, inhibit the level of URAT-1 enzyme, and then reduce the serum uric acid level of asymptomatic hyperuricemia patients (55). Animal studies have found that acupuncture at Five Shu Points of Spleen Meridian can reduce blood uric acid level by promoting uric acid excretion and increasing urine volume (56). Preclinical studies in hyperuricemia rat models have shown that acupuncture at “Shenshu” and “Taixi” points can inhibit the expression of URAT1 in the kidney of rats, thus reducing uric acid reabsorption, increasing the expression of OAT1 in the kidney, and thus reducing the level of serum uric acid (57). In hyperuricemia rat models, acupuncture at the original point and the point for raising blood can inhibit the level of xanthine oxidase, thereby reducing the production of uric acid and the level of serum uric acid in rats (58).

For symptomatic gout (acute flares), the plan prioritizes rapid analgesia and anti-inflammatory effects. Patients with gout should acupuncture Zusanli, Sanyinjiao, Quchi, Xuehai, Yanglingquan and Ashi points once a day, and at the same time cooperate with local infrared lamp irradiation, which can obviously relieve local pain and promote the recovery of normal nerve function (59). The results of metabonomics analysis show that acupuncture at Zusanli and Sanyinjiao can obviously reduce the level of serum uric acid in rats with gouty arthritis and relieve the pain in rats (60). Moreover, it has been suggested that acupuncture may have a good therapeutic effect on early gout by adjusting metabolism and improving renal function, thus reducing renal damage (61).

Electroacupuncture is the most commonly used method in acupuncture. By connecting electroacupuncture instruments to traditional acupuncture points and outputting pulse currents with different waveforms, the therapeutic effect of acupuncture can be enhanced (62). In the treatment of hyperuricemia, electroacupuncture has shown remarkable curative effect, and low-frequency (2 Hz) electroacupuncture has a better curative effect (63–65). Studies have shown that electroacupuncture stimulation of specific points, such as Zusanli and Sanyinjiao, can regulate the metabolic function of human body and promote uric acid excretion, and its mechanism may be related to improving the excretion ability of uric acid by kidney and regulating the level of inflammatory factors *in vivo* (66, 67). It is found that electroacupuncture at Zusanli, Fenglong and Ashi points can not only promote the excretion of uric acid, but also inhibit the synthesis of uric acid, thus reducing the content of sodium urate in joint tissue, thus eliminating the swelling and heat pain of patients’ joints (68). Clinical trials have demonstrated that electroacupuncture combined with diclofenac sodium significantly relieves joint pain within 10 minutes of treatment and maintains analgesic effect for up to 6 hours (69). Moreover, in patients with hyperuricemia with renal insufficiency, electroacupuncture at Shenshu, Xuehai and Sanyinjiao improves the permeability of blood on the renal cell membrane, thus restoring the uric acid excretion ability of the kidney itself (70). Animal experiments show that electroacupuncture at Zusanli and Sanyinjiao can down-regulate the expression of Cathepsin-B, thus inhibiting the activation of inflammatory corpuscles of NLRP3, reducing the expression of IL-1β and IL-18, and further inhibiting inflammatory reaction and relieving pain (71). Clinical studies have demonstrated that electroacupuncture at Zusanli and Sanyinjiao can promote the expression of Arginase-1 by activating AMPK signal pathway, induce the polarization of M2 macrophages, increase the release of anti-inflammatory cytokines, and inhibit the expression of NLRP3, thus reducing the inflammatory reaction (72). Studies have shown that electroacupuncture can also inhibit the excessive activation of NLRP3 inflammatory bodies by inhibiting the production of reactive oxygen species(ROS), and at the same time increase the up-regulation of transient receptor potential vanilloid 1(TRPV1) channels in sensory neurons to alleviate joint pain (73). In addition, it is found that electroacupuncture at Sanyinjiao, Jiexi and Kunlun points can inhibit the signal transduction of Toll-like receptor/myeloid differentiation primary response 88 (TLR/MYD88) and then the nuclear factor-κB (NF-κB) signal pathway, thus exerting anti-inflammatory effects (74). At the same time, it has been found that electroacupuncture at Sanyinjiao, Jiexi and Kunlun points can block the signal pathway of TREM-1 and reduce the inflammatory reaction (75). 2/100 Hz electroacupuncture at Zusanli and Kunlun points also significantly reduced the persistent pain behavior and ankle swelling of model rats (76).

Fire needle is an evolutionary form of acupuncture therapy in TCM. After burning a special needle, it quickly pierces the human acupoints or diseased parts. With the warm effect of high temperature, it quickly plays the roles of warming *meridians*, dispelling cold and removing dampness, reducing swelling and relieving pain (77). Fire acupuncture has a significant effect in dealing with gouty arthritis caused by hyperuricemia (78). When the fiery needle body quickly pierces the acupoints around the joint, such as Ashi point and Yanglingquan, it can quickly play the role of warming the *meridians*, dispelling cold and removing dampness, reducing swelling and relieving pain with the help of the warming effect produced by high temperature (79). Animal studies have shown that fire-needle acupuncture at Ashi points can inhibit the activation of NALP3 inflammasomes and reduce IL-1 secretion, thereby alleviating inflammation and joint swelling (80). In addition, the study found that acupuncture and moxibustion can directly act on the affected part, which can quickly improve the blood circulation around the joints, promote the absorption and dissipation of inflammatory substances, and then effectively alleviate the symptoms of redness, swelling and heat pain of the joints (81, 82).

### 3.3 Massage

As an important part of TCM therapy, massage has shown unique value and remarkable effect in the treatment of hyperuricemia and gouty arthritis caused by it (83). Massage acts on *meridians* and acupoints of human body through specific manipulations, regulating the metabolic function of the body and promoting uric acid excretion (84). Massage focuses on massaging the acupoints on the kidney meridian and bladder meridian, such as Taixi point, Shenshu point and Weizhong point, etc., to stimulate kidney function, enhance kidney’s excretion ability of uric acid, and regulate kidney’s gasification function at the same time, which is helpful to improve uric acid metabolism. Stimulating these acupoints through a series of professional massage techniques, such as kneading, pressing and pushing, can promote the smooth circulation of *qi* and *blood*, regulate the function of viscera, and thus reduce the level of blood uric acid (85). In the acute stage of gouty arthritis, massage can be done by gentle methods, such as one-finger Zen and massage, around the joints, which can effectively improve local blood circulation, promote the absorption of inflammatory substances and reduce the swelling and pain of joints (86). In the remission period of gouty arthritis, massage techniques will be aggravated appropriately, and the muscles and tendons around the joints will be massaged by rolling and pressing, so as to enhance the stability of the joints and prevent the recurrence of pain (87). Animal experimental data confirm that massage therapy can effectively inhibit the release of peripheral pain mediators K+, dopamine (DA) and norepinephrine in rats with acute gouty arthritis, thus playing an anti-inflammatory and analgesic role (88). This fully shows that massage therapy can bring positive therapeutic effect to patients with hyperuricemia and gouty arthritis, which is helpful to improve the quality of life of patients and alleviate the disease.

Although individual studies report differing technical parameters (e.g., technique definitions, operator-applied pressure, anatomical targets, symptom severity, and reporting detail), the overall operational approach can be reasonably summarized as follows and should be understood as a synthesis across multiple studies rather than a uniform protocol (42, 80, 89, 90). First, perform regional warming and tissue preparation with rolling and palm/circular gliding over peri-articular soft tissues until a gentle heat sensation is achieved (~2–3 minutes per region, intensity tailored to patient tolerance). Second, apply focused acupoint manipulation at commonly used sites for gout/gouty arthritis—Yinlingquan, Xuehai, Heding, the medial and lateral Xiyan, Zusanli, Ququan, Yanglingquan, and peri-lesional Ashi points—using press-kneading or thumb-kneading for ~1 minute per point, two sets, once daily for 7 days during the acute window. Third, use brief joint techniques as tolerated (gentle mobilization that does not provoke pain or heat; avoid vigorous manipulation directly over inflamed joints). For example, wax therapy may be applied to the affected joint at ~50 °C for 30 minutes once daily for 7 days; pricking-cupping or fire-needle bloodletting at locally engorged venules may be performed every 2 days, with a typical per-session bleeding volume ≤10 mL. Document parameters (minutes per region/point, sets, daily frequency, adjuncts), and ensure delivery by qualified clinicians to promote safety and reproducibility.

### 3.4 Bloodletting therapy

Bloodletting therapy is a TCM therapy with a long history. It uses tools such as triangular needle and plum blossom needle to release proper amount of blood at specific acupoints or superficial blood collaterals of human body, so as to regulate the circulation of *qi* and *blood* and achieve the purpose of treating diseases (91). In the field of hyperuricemia treatment, bloodletting therapy has a unique mechanism and remarkable effect (92). Based on multiple clinical studies and data-mining analyses, bloodletting sites can be broadly classified into local Ashi points and distal, specific meridian acupoints; these categories exhibit distinct profiles of efficacy with respect to lowering serum uric acid (UA) concentrations and providing analgesia.

Ashi points are the most frequently employed sites in bloodletting therapy. By definition, the puncture target is the locus of maximal joint erythema, swelling, warmth, and pain (93), embodying the therapeutic principle of “treat where it hurts” (yi tong wei shu). Contemporary studies indicate that Ashi-point bloodletting not only modulates inflammatory signaling pathways and reduces inflammatory cell infiltration (94), but also suppresses the release of peripheral pain mediators (e.g., K^+^, 5-hydroxytryptamine (5-HT), dopamine), thereby effectively alleviating swelling and pain—a profile particularly suitable for acute gout flares (95). By contrast, meridian acupoint bloodletting exerts more pronounced systemic regulatory effects. In gouty arthritis, commonly selected points include Taichong (LR3), Xingjian (LR2), Neiting (ST44), Sanyinjiao (SP6), Xuehai (SP10), Yinlingquan (SP9), and Weizhong (BL40), distributed along the Liver, Spleen, Stomach, Gallbladder, and Bladder *meridians*. These acupoints may improve whole-body metabolic status via soothing the Liver and regulating *qi*, clearing heat and draining dampness, and invigorating blood to unblock collaterals. Among them, Taichong and Xingjian can dissipate excess heat from the Liver meridian; Xuehai and Yinlingquan promote blood circulation and facilitate uric acid excretion; and Sanyinjiao may modulate vasomotor tone and relieve pain (96, 97). Weizhong (BL40), a He-Sea point of the Foot Taiyang Bladder meridian, is traditionally indicated for dispelling dampness, resolving stasis, and relieving pain. Clinically, Weizhong bloodletting combined with cupping plus febuxostat achieved an overall response rate of 93.33%, significantly higher than 63.33% with febuxostat alone; this combined regimen lowered serum uric acid (SUA) and C-reactive protein (CRP), improved joint mobility and pain, and likely acted by enhancing microcirculation and promoting urate excretion, thereby delivering an integrated urate-lowering and anti-inflammatory/analgesic effect (35). These observations suggest that meridian-point bloodletting not only contributes to analgesia but also facilitates meridian–viscera regulatory pathways that support urate homeostasis, making it suitable for remission or subacute management.

Beyond limb acupoints, auricular sites (e.g., the auricular helix) show unique value for urate reduction. A clinical study reported that auricular pricking bloodletting plus auricular seed pressing combined with dietary counseling produced significantly lower SUA levels than dietary counseling alone, indicating that auricular bloodletting—as a distal, specialized approach—has distinctive advantages in modulating systemic urate metabolism (98).

In summary, convergent evidence indicates that site selection materially influences clinical outcomes: local Ashi-point bloodletting primarily affords rapid analgesia for acute symptoms, whereas distal/meridian (including auricular) bloodletting emphasizes longer-term urate regulation. A combined strategy may produce synergistic benefits in both pain control and urate lowering (35).

Dose and frequency parameters. In current clinical practice, there is no unified quantitative standard for the volume of bloodletting, which renders therapeutic outcomes uncertain. In general, insufficient volume removes too few pro-inflammatory mediators and fails to produce adequate microcirculatory benefits, resulting in suboptimal efficacy; conversely, excessive volume may “injure essence and deplete *qi*,” raising safety concerns. Published clinical protocols report a wide per-session range that depends on technique and disease severity: approximately 2–10 mL for mild presentations, with 30–60 mL reported in selected severe cases. Several comparative cohorts have explored graded single-session doses of 20/40/60 mL and observed a dose–response relationship, i.e., greater improvement in gouty-arthritis symptoms and larger reductions in UA at higher volumes (99). Accordingly, we propose a routine cap of ≤ 10–20 mL for localized venous bloodletting, and an upper limit of 30–50 mL when systemic manifestations are prominent and close clinical monitoring is ensured.

Bloodletting frequency also varies across studies. For asymptomatic or mild cases, a typical schedule is once per week. For acute, severe flares, several clinical protocols perform sessions every 3–4 days (i.e., ≤i sessions/week) as a short course (typically three sessions), followed by clinical reassessment (99, 100).

In summary, the choice of bloodletting volume and frequency should be closely individualized to the patient’s constitution and disease severity. Moreover, bloodletting should be performed by qualified clinicians, with per-session volume and weekly frequency strictly regulated and documented to ensure treatment safety and effectiveness.

### 3.5 Topical Chinese herbal therapy

Topical Chinese herbal therapy is a kind of external therapy of TCM. These drugs penetrate through the skin, run along the *meridians* and *qi* and *blood*, and play a role in treating diseases (101). Ttai (102, 103). It is found that Luo Qing powder (Qingluo San), a topical herbal preparation typically composed of *Sinomenium acutum* (*Caulis Sinomenii*), *Stephania tetrandra* (*Radix Stephaniae tetrandrae*), *Artemisia anomala* (*Herba Artemisiae Anomalae*), and *Atractylodes lancea* (*Rhizoma Atractylodis*), combined with diclofenac sodium, can relieve the joint swelling and pain of patients with acute gouty arthritis more quickly and improve the clinical efficacy. The total effective rate is as high as 96.55% (104). External therapy of compound Qingbi granule, which often includes herbs such as *Smilax glabra* (*Rhizoma Smilacis Glabrae*), *Angelica pubescens* (*Radix Angelicae Pubescentis*), *Saposhnikovia divaricata* (*Radix Saposhnikoviae*), and *Ligusticum chuanxiong* (*Rhizoma Ligustici*), can quickly relieve swelling and pain, prolong the time of pain relief and inhibit inflammatory reaction, and there is no obvious adverse reaction at the same time (105). In addition, the research shows that topical hot compress with Sihuangshuimi, a decoction containing *Coptis chinensis* (*Rhizoma Coptidis*), *Scutellaria baicalensis* (*Radix Scutellariae*), *Phellodendron chinense* (*Cortex Phellodendri*), and *Gardenia jasminoides* (*Fructus Gardeniae*), can dilate local blood vessels, improve local blood circulation, increase metabolism, and make drugs penetrate and absorb through the skin and directly enter the lesion, thus playing a therapeutic role (106). Xinhuang patch, a transdermal formulation composed of herbal ingredients such as *Commiphora myrrha* (*Myrrha*), *Boswellia carterii* (*Olibanum*), *Borneolum* (*Borneolum Syntheticum*), and *realgar* (*Arsenicum*), may improve joint pain and swelling in patients with gouty arthritis by reducing C-reactive protein and erythrocyte sedimentation rate (107). Moreover, the external therapy of Tibetan medicine Qingpeng Ointment, which contains herbs such as *Aconitum kusnezoffii* (*Radix Aconiti Kusnezoffii Preparata*), *Ephedra sinica* (*Herba Ephedrae*), *Angelica dahurica* (*Radix Angelicae Dahuricae*), and *Rheum palmatum* (*Radix et Rhizoma Rhei*), has been shown to be effective in improving joint pain and dysfunction of patients with gouty arthritis, except for skin irritation in some patients, and there is no obvious adverse drug reaction (108, 109). Most importantly, topical Chinese herbal therapy the application as an external therapy method avoids the irritation of oral drugs to the gastrointestinal tract, reduces the adverse drug reactions, and provides a safe and effective green treatment option for patients with hyperuricemia and gout (110). A summary of clinical and preclinical evidence for various external therapies of TCM in the treatment of hyperuricemia is presented in **Table 1**.

**Table 1:** Traditional Chinese medicine external therapy for hyperuricemia.

Therapy	Study type	Subjects	Site of action	Main observed effects	References
Acupuncture	Meta-analysis	—	—	Pain ↓	(51, 52)
	Clinical trial	Patients	Five shu acupoints	Uric acid excretion. Blood uric acid level ↓	(56)
	Clinical trial	Patients	Zusanli, Sanyinjiao, Quchi, Xuehai, Yanglingquan, Ashi acupoint	Paino Blood uric acid level ↓	(59)
	Meta-analysis	—	—	Uric acid productions Uric acid excretion ↑	(54)
	Clinical trial	Patients	Five shu acupoints	URAT1i Urine PHi Blood uric acid level ↓	(55)
	Animal experiments	Male SD rats	Zusanli, Sanyinjiao	Paini Blood uric acid level ↓	(60)
	Animal experiments	Male Wistar rats	Shenshu, Taixi	URAT1h OAAT1h Blood uric acid level ↓	(57)
	Animal experiments	Male Wistar rats	Yuan points, Mu points	XODn Blood uric acid level ↓	(58)
	Clinical trial	Patients	Ashi acupoint	Paino Blood uric acid level ↓	(64)
	Clinical trial	Patients	Zusanli, Sanyinjiao, Ashi acupoint	Paino Blood uric acid level ↓	(65)
	Clinical trial	Patients	Zusanli, Sanyinjiao, Fenglong, Yinlingquan, Ashi acupoint	Pain ↓	(66)
	Clinical trial	Patients	Zusanli, Sanyinjiao, Kunlun, Taixi, Xuehai, Yanglingquan, Ashi acupoint	Pain ↓	(67)
	Clinical trial	Patients	Zusanli, Fenglong, Ashi acupoint	Uric acid productionC Uric acid excretionn Pain ↓	(68)
	Clinical trial	Patients	Zusanli, Sanyinjiao, Taichong, Yinlingquan	Pain ↓	(69)
	Clinical trial	Patients	Fenglong, Sanyinjiao, Yinlingqyuan	Uric acid productiona Uric acid excretionn Pain ↓	(70)
	Animal experiments	Male wistar rats	Zusanli, Sanyinjiao	Pain ↓	(71)
	Animal experiments	Male wistar rats	Zusanli, Sanyinjiao	Uric acid productions Uric acid excretionn Pain ↓	(72)
	Animal experiments	Male C57BL/6J mice	Ashi acupoint	Pain ↓	(73)
	Animal experiments	Male SD rats	Sanyinjiao, Jiexi, Kunlun	Uric acid production, Uric acid excretionn Pain ↓	(74, 75)
	Animal experiments	Male SD rats	Zusanli, Kunlun	Pain ↓	(76)
	Clinical trial	Patients	Yanglingquan, Ashi acupoint	Pain ↓	(79)
	Animal experiments	Male wistar rats	Ashi acupoint	Pain ↓ Swelling ↓	(57)
Massage	Clinical trial	Patients	Ashi acupoint	Pain ↓ Swelling ↓	(45)
	Clinical trial	Patients	Taixi, Shenshu, Weizhong	Pain ↓ Swelling ↓	(90)
	Clinical trial	Patients	Ashi acupoint	Pain ↓ Swelling ↓	(83)
	Animal experiments	Male SD rats	Ashi acupoint	Pain ↓	(88)
Bloodletting therapy	Clinical trial	Patients	Ear points	Blood uric acid level ↓	(46)
	Clinical trial	Patients	Ashi acupoint	Pain ↓	(35)
	Clinical trial	Patients	Ashi acupoint	Pain ↓	(88)
	Animal experiments	Male SD rats	Ashi acupoint	Pain ↓	(95)
	Clinical trial	Patients	Ashi acupoint	Pain ↓	(91)
	Clinical trial	Patients	Weizhong (BL40)	Pain ↓	(111)
	Clinical trial	Patients	Painful area	Pain ↓, Metabolism ↑	(106)
	Clinical trial	Patients	Painful area	Pain ↓	(108)
	Clinical trial	Patients	Painful area	Pain ↓, Dysfunction of joint function ↓	(108, 109)
	Meta-analysis	——	——	Pain ↓, Adverse drug reactions ↓	(110)

Note: ↑ = increased; ↓ = decreased. SUA = Serum uric acid; CRP = C-reactive protein; ESR = Erythrocyte sedimentation rate. URAT1 = Uric acid transporter 1; OAT1 = Organic anion transporter 1; ROS = Reactive oxygen species; TRPV1 = Transient receptor potential vanilloid 1. Acupoints are labeled using WHO standard nomenclature.

## 4 Prospects for the future

The pathogenesis of hyperuricemia is complex, involving the interaction of many factors, including genetic factors, eating habits, lifestyle and endocrine disorders (2). In modern medicine, although drug therapy has achieved certain results, there are still some problems such as side effects, poor compliance and the impact on the overall health of patients (112). At the same time, the external therapy of TCM has shown remarkable effectiveness and potential in the treatment of hyperuricemia, which has been paid more and more attention by researchers and clinicians (113). Through the comprehensive application of acupuncture, massage, bloodletting and topical Chinese herbal therapy, the clinical manifestations and quality of life of patients with hyperuricemia can be improved in many ways (87). The most important thing is that the external therapy of TCM emphasizes individualized treatment, which conforms to the principle of “syndrome differentiation and treatment” of TCM, and can better meet the needs of patients, adjust their physical condition, relieve pain, improve blood circulation and other ways, effectively reduce the discomfort of patients and improve the effectiveness of treatment.

Recent studies suggest that targeting the gut microbiota offers a promising strategy for managing hyperuricemia (114). By modulating the microbiota, plant-derived natural products can reduce uric acid (UA) levels through purine degradation, reduced UA production, and enhanced excretion. These compounds also have anti-inflammatory and antioxidant effects, alleviating complications like renal damage (114). This approach could provide a natural, safer alternative or adjunct to conventional treatments for hyperuricemia.

Integrative Chinese–Western management of gout and hyperuricemia generally demonstrates favorable efficacy and safety. Given that some patients have concerns about the tolerability and safety of long-term Western pharmacotherapy, and that external therapies of TCM show potential to improve symptoms and quality of life, integrative regimens may offer a pathway with greater accessibility and patient acceptability in clinical practice (115, 116). Specifically, two complementary collaborative approaches may be considered. First, a phase-based pathway: during acute flares, anchor care to Western anti-inflammatory analgesia (e.g., NSAIDs, colchicine, or short-course glucocorticoids) while concurrently applying external therapies of TCM—acupuncture, massage, bloodletting, and topical preparations—to further enhance pain relief, reduce swelling, and restore function; during the intercritical/remission phase or in isolated hyperuricemia, maintain urate-lowering therapy as the foundation and overlay external therapies of TCM to support metabolic modulation and prevent flares, thereby achieving a “system control + symptom management” synergy. This approach aligns closely with integrated management advocated in recent combined Chinese–Western guidelines/consensus statements (117). Second, a pattern-guided collaborative plan: use Western therapy as the principal disease-modifying “axis” for urate and inflammation control, and match external therapies of TCM according to common TCM patterns (e.g., Damp-Heat, Phlegm-Turbidity, Blood-Heat), such as acupuncture to clear Heat and drain Dampness, tuina to free the channels and invigorate Blood, and—when appropriate and within safety limits—bloodletting; dosing and frequency are then fine-tuned within predefined ranges based on individual response to balance evidence comparability with personalization (118).

Nevertheless, challenges remain. Standardization and normalization of technical parameters for external therapies of TCM (e.g., frequency, dose, and course length) are still limited, leading to substantial heterogeneity across studies and constraining comparability and verification of efficacy. Moreover, although existing studies suggest that these therapies can alleviate pain and swelling and lower serum urate, many involve small samples and lack large, multicenter randomized trials, which weakens the robustness and generalizability of conclusions. In addition, rigorously designed, head-to-head pharmacoeconomic evaluations comparing the cost–effectiveness of TCM external therapies and Western pharmacotherapy are currently lacking. Accordingly, we call for more high-quality, multicenter clinical and mechanistic studies with standardized reporting of treatment parameters to systematically evaluate the efficacy, safety, and cost-effectiveness of external therapies of TCM in hyperuricemia—and to advance replicable, scalable Chinese–Western collaborative pathways.

Furthermore, despite the growing interest and preliminary evidence supporting the efficacy of external TCM therapies in managing hyperuricemia, the translation of these innovations into routine practice remains limited. Several barriers hinder broader clinical adoption, including lack of standardization in intervention protocols (e.g., frequency, dosage, and duration), heterogeneity in study designs, and insufficient reporting of treatment parameters (119, 120). Notably, some studies have reported inconsistent or non-significant effects on serum uric acid reduction, especially in trials with small sample sizes or inadequate control groups, suggesting that the benefits of external therapies may not be universally reproducible (121). In addition, although adverse events are generally mild, potential risks still exist: improper fire-needle manipulation may cause local burns or infection (122); excessive bloodletting may lead to dizziness, anemia, or delayed wound healing; and certain topical herbal preparations can induce contact dermatitis or allergic reactions, particularly in individuals with sensitive skin (111, 123). These contradictory findings and safety concerns further highlight the need for rigorous monitoring and standardized safety procedures. Additional challenges include variable practitioner training, insufficient integration with Western care models, and the absence of pharmacoeconomic evaluations comparing TCM external therapies with conventional pharmacological approaches. Collectively, these limitations underscore the urgent need for multicenter, high-quality randomized controlled trials, long-term safety assessments, and standardized clinical guidelines to support evidence-based and safe implementation of innovative external TCM therapies for hyperuricemia.

## 5 Conclusion

Hyperuricemia, as an increasingly common metabolic disease, has brought many troubles to patients’ quality of life and health. In recent years, with its unique advantages, external therapy of TCM has emerged in the treatment of hyperuricemia, showing a broad application prospect. external therapy of TCM has played an active role in improving patients’ symptoms, reducing blood uric acid level and reducing complications by stimulating acupoints, regulating *qi* and *blood* and promoting uric acid excretion. Its characteristics of small side effects and overall adjustment of body functions make it an important supplementary means for the treatment of hyperuricemia. Current evidence indicates that external therapies of TCM have a significantly lower incidence of adverse events (AEs) than Western pharmacotherapies. For example, in a meta-analysis of 9 RCTs (n = 735) evaluating bloodletting, the AE rate was 2.1% (8/383) in the bloodletting arm versus 9.09% (32/352) with conventional Western therapy (RR = 0.224, 95% CI [0.110–0.453]; Z = 4.16; p<0.001; I² = 0%), indicating superior overall tolerability (35). In addition, two further meta-analyses reported similarly lower AE rates with bloodletting—predominantly local, mild, and reversible events—thereby supporting the safety advantage of external TCM therapies in terms of fewer systemic adverse reactions (35, 87). Likewise, in a meta-analysis of acupuncture for acute gouty arthritis that included 6 RCTs reporting AEs, the AE rate was 2.1% (5/239) with acupuncture versus 28.0% (61/218) with Western medication, further supporting the conclusion that external therapies have fewer side effects and that AEs are mainly local and mild (94).

According to the clinical research and experimental research in recent years, we summarized the application of external Chinese medicine therapy including acupuncture, massage, bloodletting and Chinese medicine application in hyperuricemia and gouty arthritis caused by hyperuricemia (**Table 1**), in order to provide detailed theoretical basis for the subsequent clinical application of external Chinese medicine therapy in hyperuricemia.

In a word, the external therapy of TCM has important implementation and popularization significance in the clinical practice of hyperuricemia. It not only provides patients with safe and effective treatment options, but also improves their quality of life and prevents the occurrence and recurrence of diseases through individualized treatment strategies. In the future, with the in-depth study of TCM external therapy, TCM external therapy will play a greater role in the treatment of hyperuricemia by continuously deepening theoretical research, actively promoting medical technology innovation, continuously expanding clinical application, and strengthening the research of related treatment mechanisms.
